# Effect of Octreotide on Lymphorrhea in Patients After Modified Radical Mastectomy for Carcinoma Breast: A Randomized Controlled Trial

**DOI:** 10.7759/cureus.19225

**Published:** 2021-11-03

**Authors:** Sahaj Prajapati, Sadhasivam Ramasamy, Manu Vats, Sushanto Neogi, Ketan Kantamaneni, Sanjeev Kumar Tudu

**Affiliations:** 1 Pediatric Surgery, Lady Hardinge Medical College, New Delhi, IND; 2 Surgery, Milton Keynes University Hospital, Milton Keynes, GBR; 3 Surgery, Maulana Azad Medical College, New Delhi, IND; 4 Surgery, Dr. Pinnamaneni Siddhartha Institute of Medical Sciences and Research Foundation, Gannavaram, IND; 5 Surgery, Lady Hardinge Medical College, New Delhi, IND

**Keywords:** cancer, octreotide, seroma, lymphorrhea, modified radical mastectomy, carcinoma, breast

## Abstract

Introduction

Lymphorrhea or seroma formation after modified radical mastectomy (MRM) is a serious and disabling complication of axillary lymphadenectomy. Octreotide is a hormone with general anti-secretory effects. The potential role of octreotide in the treatment of lymphorrhea after axillary lymph node dissection in patients undergoing MRM is being investigated in this study. The purpose of this research is to study the effect of octreotide on the magnitude and duration of lymphorrhea in patients after MRM for carcinoma breast.

Methods

This clinical trial was registered in the Clinical Trials Registry India (CTRI/2017/11/010653). It was conducted in the Department of General Surgery, Maulana Azad Medical College and associated Lok Nayak Hospital, New Delhi from September 2015 to March 2017. This study is a parallel randomized controlled trial with a 1:1 allocation ratio. Thirty patients were enrolled and allocated equally into two groups. The intervention group received standard medical care plus injection octreotide 100 micrograms eight hourly intravenously post-operatively for five days and the control group received only standard medical care. The primary outcomes were lymphorrhea volume from 24 hours post-surgery till five days post-operatively and the number of days till the suction drain was removed. Secondary outcomes were surgical site infection, the incidence of seroma formation, complications of octreotide, duration of hospital stay, and the number of lymph nodes isolated. All the patients were followed up twice a week for the first six weeks after discharge followed by three monthly visits.

Results

A total of 30 patients were included in the study. The mean age was 46.2 years. The mean operative time in the control group was 137.87 ± 23.28 minutes and in the octreotide group was 128.13 ± 12.29 (p = 0.163). The volume of lymphorrhea in the control group was 354.67 ± 346.28 ml and in the octreotide group was 194.00 ± 240.62 ml (p = 0.081). Seroma occurred in 9% of patients in the control group and 2 % of patients in the octreotide group (p = 0.010). The duration of lymphorrhea was 4.93 ± 2.49 days in the control group and 3.13 ± 1.36 days in the octreotide group (p = 0.029). The duration of stay was 7.07 ± 2.40 days in the control group and 5.13 ± 1.06 days in the octreotide and was found to be statistically significant (p = 0.010). No obvious adverse reactions related to injection octreotide, namely, nausea, vomiting, abdominal discomfort, hypotension, bradycardia, and dysglycemia, were seen in any of our patients.

Conclusion

The duration of lymphorrhea, incidence of seroma formation, and duration of hospital stay were lesser in the octreotide group, and the difference was statistically significant. The wound infection rates were similar in both groups. Thus injection octreotide can be used safely and effectively.

## Introduction

Breast carcinoma is the most common cancer in women and contributes to 22.9% of all cancers, excluding non-melanoma skin cancers [1]. In developing countries, more than 90% of the cases are diagnosed when the disease reaches advanced stages, i.e., stages IIB, III, and IV [2]. Breast cancer management has evolved significantly over the years, from the radical extirpation of the whole breast, skin, and underlying muscle practiced in the previous century, to the breast conservation approach in today’s current practice [3].

Despite the emergence of breast conservation surgery (BCS), modified radical mastectomy (MRM) remains the most commonly performed surgery for breast cancer today [4]. Earlier trials have demonstrated equivalent long-term survival rates for patients with early-stage invasive breast cancer treated by mastectomy or BCS [5]. Axillary lymph node dissection for local control of nodal disease is an integral part of MRM.

Lymphorrhea or seroma formation is a serious and disabling complication of axillary lymphadenectomy, but no effective therapy is currently available [6]. Lymphorrhea adds to morbidity in the form of prolonged drainage and can also significantly impact treatment by delaying adjuvant therapy and increasing the risk for infection [7]. Although the exact pathogenesis of seroma formation has not yet been determined, multiple incriminating factors have been established [8]. Once dissection is done, the empty space is replaced with a serous fluid [9]. This fluid undergoes serial changes in its composition over the period of time following the surgical procedure; initially, it has lymph and blood clots, and later on, it becomes exudative in nature [10]. This "seroma" then prevents and delays the apposition of the skin flaps to the chest wall, thereby delaying the wound healing process [11,12]. The commencement of active upper-limb physiotherapy further results in the oozing of damaged lymphatic and blood vessels [13]. In some cases, persistent lymphorrhea may require a re-operation. A delayed complication of lymphorrhea is the development of lymphosarcoma of the upper limb. This has been linked to the duration and amount of lymphorrhea [14]. Various techniques for the reduction of lymphorrhea have been tried in the past but with limited success. These include fibrin glue application, suturing skin flaps with underlying tissue, lanreotide autogel, tranexamic acid, and suction drain placement [15].

Octreotide is a hormone with general anti-secretory effects and has been classically used in the treatment of portal hypertension, oesophageal variceal bleed, VIPoma, carcinoid tumors, and pancreatic fistulas. Common adverse events noted after using octreotide are gallbladder dyskinesia, cholestatic hepatitis, dysglycemia, hypothyroidism, and bradycardia. Octreotide has also been tried in controlling lymphorrhea in thoracic duct injury, after radical neck dissection and pelvic lymph node dissection [16].

Based on this knowledge, the potential role of octreotide in the treatment of lymphorrhea after axillary lymph node dissection in patients undergoing MRM can be investigated.

The purpose of this research is to study the effect of octreotide on the magnitude and duration of lymphorrhea in patients undergoing MRM.

## Materials and methods

This study was conducted in the Department of General Surgery, Maulana Azad Medical College and associated Lok Nayak Hospital, New Delhi from September 2015 to March 2017. All the adult female patients who underwent MRM for locally advanced breast carcinoma and gave voluntary consent were included in the study. Patients with previous irradiation to breast, hepatic and renal impairment, cardiac disease, and patients with hypersensitivity to octreotide were excluded from the study. The study protocol was approved by the institute’s ethical committee. Written informed consent was taken from all the participating patients.

The sample size was calculated and came out to be 50 in each group; however, due to restrictions of time and subjects, a total of 30 patients were studied.

Initially, 34 patients were assessed for eligibility. However, four patients were excluded as three patients refused to give consent for participation and one patient did not meet the inclusion criteria (had a cardiac disease). Eventually, a total of 30 patients were included in this study and were randomized into two groups using a computer-generated sequence. The generated sequence (group A or B) was entered separately and sequentially on slips of paper, which were folded and thereafter numbered till 30. The postgraduate surgery resident enrolled the participants and assigned their respective intervention groups. Group A included 15 patients and received standard medical care without injection octreotide whereas group B included 15 patients and received standard medical care with injection octreotide after surgery in the post-operative period. This study is a parallel randomized controlled trial with a 1:1 allocation ratio.

**Figure 1:**
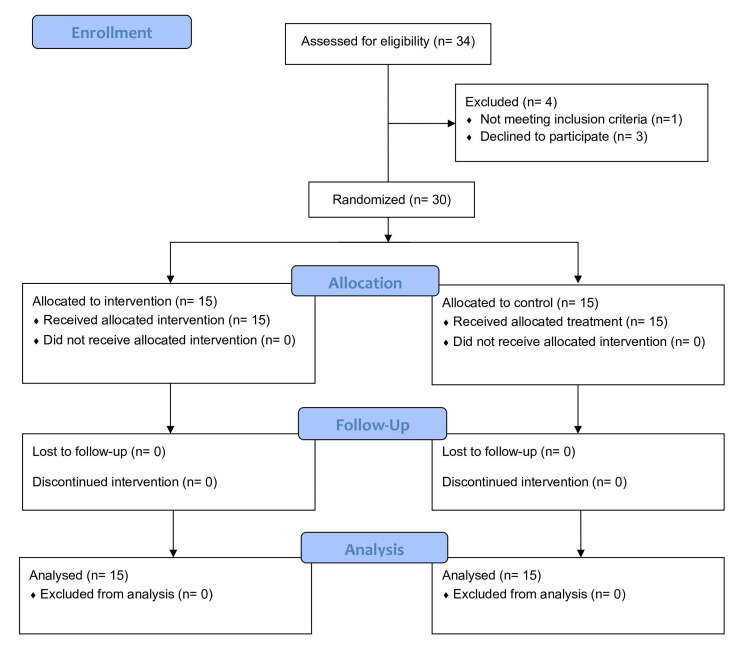
CONSORT flow diagram. CONSORT, Consolidated Standards of Reporting Trials.

All the eligible patients underwent standard MRM with axillary node dissection under general anesthesia. A closed suction drain with two limbs was placed; one limb was placed over the chest wall, under the skin flaps, and the other limb in the axilla. The limbs were removed when the daily drain output was less than 50 ml. Post-operatively, axillary compression was given to both groups using crepe bandage for the first 24 hours. Day of surgery was defined as day 0. Patients were randomized into groups A and B by computer-generated random number sequence. Group A received standard care for MRM. Group B received injection octreotide 100 micrograms eight hourly (after sensitivity testing) for five days. The Consolidated Standards of Reporting Trials (CONSORT) flow diagram is shown in Figure 1.

The primary outcomes were lymphorrhea volume from 24 hours to five days post-operatively, and the number of days till the suction drain was removed. Secondary outcomes were surgical site infection, the incidence of seroma formation, complications of octreotide, duration of hospital stay, and the number of lymph nodes isolated. All the therapeutic aspirations are done for post-operative fluid collection, which was more than 50 ml counted as seroma formation. All the wound infections and flap necrosis were treated by aseptic debridement and dressings. All the patients were followed up twice a week for the first six weeks after discharge followed by three monthly visits.

Ethics

The study protocol was approved by the Institutional Ethics Committee of Maulana Azad Medical College (F.No./11/IEC/MAMC/2015/317). All procedures performed in studies involving human participants were in accordance with the ethical standards of the institutional and/or national research committee and with the 1964 Helsinki declaration and its later amendments or comparable ethical standards. This research did not receive any specific grant from funding agencies in the public, commercial, or not-for-profit sectors.

Statistics

Statistical analysis was done using SPSS version 17.0. Results were expressed as mean ± standard deviation and number (%). The categorical variables were compared using the chi-square test, Fisher’s exact test, unpaired Student's t-test, and independent samples Mann-Whitney U-test. A p-value (two-tailed) <0.05 was taken as significant.

## Results

Demography

A total of 30 patients were included in the study and all the patients underwent standard MRM. The range of age was between 20 and 70 years with the mean age being 46.2 years. Out of the 30 patients, mostly were seen to lie in the range of 30-50 years, and the difference was not found significant statistically between groups A and B. The results of the study are shown in Table 1.

**Table 1:** Results of the study. SD, standard deviation; * significant p-value.

Parameter	Control group A (n = 15)	Octreotide group B (n = 15)	p-value
Age in years (mean ± SD)	44.86 ± 10.21	47.53 ± 13.81	0.553
Operative time in minutes (mean ± SD)	137.87 ± 23.28	128.13 ± 12.29	0.163
Volume of lymphorrhea in milliliters (mean ± SD)	354.67 ± 346.28	194.00 ± 240.62	0.081
Duration of lymphorrhea in days (mean ± SD)	4.93 ± 2.49	3.13 ± 1.36	0.029*
Duration of hospital stay in days (mean ± SD)	7.06 ± 2.40	5.13 ± 1.06	0.010*
Daily drain output in milliliters (mean ± SD)	92.43 ± 46.83	105.28 ± 51.83	0.389
Wound infection (%)	2 (13.6)	0 (0)	0.483
Flap necrosis (%)	3 (20)	0 (0)	0.224
Seroma formation (%)	9 (60)	2 (13)	0.010*
Total number of lymph nodes (mean ± SD)	12 ± 4.48	15.27 ± 5.29	0.116
Amount of lymphorrhea per lymph node (mean ± SD)	23.77 ± 29.60	14.47 ± 20.76	0.050
Number of positive lymph nodes (mean ± SD)	0.53 ± 0.83	1.33 ± 1.72	0.250
Amount of lymphorrhea by positive lymph nodes (mean ± SD)	138.00 ± 261.074	29.67 ± 35.15	1.000

Operative time

Operative time was calculated as the time from the first skin incision to the last skin staple/stitch. The control group had a mean operative time of 137.87 ± 23.28 minutes and the octreotide group had a mean operative time of 128.13 ± 12.29 minutes and the p-value was 0.163.

Volume of lymphorrhea

The volume of lymphorrhea was calculated by excluding drain output of the first 24 hours from total drain output since it was considered to be serosanguineous fluid after dissection. It was one of the primary outcomes of the study. The volume of lymphorrhea in the control group was 354.67 ± 346.28 ml and in the octreotide group was 194.00 ± 240.62 ml. Though, the difference in the volume of lymphorrhea in the octreotide group and control group was not significant statistically (p-value = 0.081); nevertheless, it was almost half in the octreotide group as compared to the control group.

Duration of lymphorrhea

The drain was removed once the drain output decreases below 50 ml. The total number of days till the drain removal was done was considered as the duration of lymphorrhea. It was 4.93 ± 2.49 days in the control group and was 3.13 ± 1.36 days in the octreotide group and was statistically significant (p = 0.029).

Duration of hospital stay

The duration of the post-operative hospital stay was counted from the day of surgery till the patient was discharged from the hospital in days. The duration of stay was 7.07 ± 2.40 days in the control group and 5.13±1.06 days in the octreotide and was found to be statistically significant (p = 0.010).

Daily drain output

Daily drain output was calculated by dividing total drain output by the number of days to drain removal. It was 92.4 ± 46.83 ml in the control group and 105.28 ± 51.83 ml in the octreotide group and was not statistically significant (p = 0.389).

Seroma formation

Seroma formation was defined as a serous fluid collection under the skin flap due to dissected lymphatics and blood vessels during surgery with serous exudate from the stitch line. Seromas which needed intervention (aspiration > 50 ml) were taken into consideration. It was 9% in the control group and 2 % in the octreotide group and was statistically significant (p = 0.010).

Wound complications

Wound complications like wound infections and flap necrosis were higher in the control group but were not statistically significant. In our study, only two (13.6%) patients in the control group and none in the octreotide group had wound infection. Flap necrosis was noticed in three (20%) patients in the control group and none in the octreotide group. None of our patients developed a hematoma, ecchymosis, and other complications.

Total number of lymph nodes

The total number of lymph nodes removed was compared in group A and group B to examine the hypothesis that the number of lymph nodes removed can influence the amount of lymphorrhea and so total drain output. The mean number of lymph nodes removed was higher in the octreotide group (15.27 ± 5.29) than in the control group (12 ± 4.48). The difference between the two groups was not significant statistically (p = 0.116).

Volume of lymphorrhea per lymph node

The volume of lymphorrhea divided by the total number of lymph nodes removed was considered as the volume of lymphorrhea per lymph node and was compared in the two groups. It was 23.77 ± 29.60 ml in the control group and 14.47 ± 20.76 ml in the octreotide group but was not statistically significant (p = 0.050).

Number of positive lymph nodes

The number of positive lymph nodes was compared to assess the influence on the volume of lymphorrhea. The mean number of positive lymph nodes was higher in the octreotide group (1.333 ± 1.72) than in the control group (0.533 ± 0.83). The difference in both groups was not significant statistically (p =0.250). Hence, the number of positive lymph nodes was comparable in both groups.

Volume of lymphorrhea by positive lymph nodes

The volume of lymphorrhea divided by the number of positive lymph nodes was considered as the amount of lymphorrhea by positive lymph nodes and was compared in both groups. It was 138.00 ± 261.07 ml in the control group and 29.666 ± 35.15 in the octreotide group but was not found to be statistically significant (p = 1.00).

Complications of octreotide

No obvious adverse reactions related to injection octreotide, namely, nausea, vomiting, abdominal discomfort, hypotension, bradycardia, and dysglycemia, were seen in any of our patients.

## Discussion

Axillary dissection is associated with complications such as wound infection, lymphedema, prolonged lymphorrhea, and fluid collection (seroma). Prolonged lymphorrhea and seroma formation are the most common complications after breast cancer surgery. The exact etiology of seroma formation has still not been understood completely. Several interventions have been tried in the past to decrease seroma formation. These include ultrasonic shears for lymphadenectomy, fibrin glue, buttress suture, fibrin sealant, suction drain placement, and octreotide injection [7,14,17-19].

Upon reviewing the available literature, we came across very few similar research studies which have investigated the role of injectable octreotide in reducing lymphorrhea. A prospective randomized controlled trial, carried out by Carcoforo et al., enrolled 261 patients who underwent axillary lymph node dissection. The "test" group, consisting of 125 patients, received 0.1 mg of injection octreotide subcutaneously three times for five days post-operatively. The authors observed that the test group had a significant reduction in post-operative seroma formation and duration. Furthermore, this group had a lower number of post-operative wound infections [6]. This result was in accordance with the observation that the patients having the lowest volumes of oedema fluid are more likely to recover successfully with minimal morbidity [20]. The authors concluded that octreotide can be used with success in controlling the amount of lymphorrhea and thereby reducing patient morbidity [6].

In another novel study, the authors investigated the role of somatostatin on the duration of lymphorrhea in female patients who underwent MRM with level II axillary lymph node dissection. A total of 50 patients were divided into two groups; the test group consisted of 30 patients, who received somatostatin 0.1 mg subcutaneously every eight hours for seven days and the control group consisted of 20 patients who received no somatostatin. The results confirmed that the group which received octreotide had a statistically significant (p = 0.0001) less duration of lymphorrhea as compared to the control group. Octreotide use would not only reduce the duration of lymphorrhea but also reduce the hospital stay period, improve patient's life quality and enable early administration of adjuvant treatment [14].

Octreotide use has also been extended to other surgical procedures to reduce post-operative lymphorrhea. In one such study, the authors established the efficacy and safety of octreotide for the management of lymphorrhea after pelvic lymph node dissection in radical prostatectomy. The patients that were treated with octreotide had significantly reduced lymphorrhea, early drain removal, and a reduced duration of hospital stay in comparison to the untreated group. Octreotide use was not associated with any significant side effects. Thus, the researchers concluded that octreotide injection is a safe and effective therapy for the management of post pelvic lymph node dissection lymphorrhea [21]. The role of the drug in reducing "lymphorrhagia" after thoracic duct rupture was also highlighted in an earlier article [22].

In the present research, a randomized controlled study was done to study the efficacy of octreotide in decreasing the volume and duration of lymphorrhea. The study compared the effect of octreotide on the volume and duration of lymphorrhea after MRM. The following results were obtained from the study. First, the volume of lymphorrhea was almost half in the octreotide group as compared to the control group but was not statistically significant. Hence, it can be concluded that octreotide has some effect on decreasing the amount of lymphorrhea but not of statistical significance. However, the duration of lymphorrhea was lesser in the octreotide group than in the control group and was statistically significant. Subsequently, the duration of hospital stay was lesser in the octreotide group than in the control group and was found to be statistically significant. The wound infection rates were similar in both groups; therefore, octreotide use does not increase any risk of wound complications. Furthermore, there was no incidence of flap necrosis in the octreotide group. The incidence of seroma formation in the octreotide group was significantly lower than in the control group. The number of positive lymph nodes and duration of the operative procedure in both groups were also comparable. Finally, the amount of lymphorrhea by positive node was higher in the control group but the difference was not significant.

Other interventions to decrease post-operative lymphorrhea have been described in the literature. The fixation of flaps to the anterior chest wall muscles using sutures obliterates the dead space created after MRM. This additive procedure is associated with a significant reduction in the post-operative seroma formation and duration of lymphorrhea. Another conclusion that the researchers made was that irrespective of the drain output, the removal of drains on post-operative day seven after MRM did not increase the chances of seroma formation. However, operative site chest wall compression dressings did not reduce the lymphorrhea significantly [23]. It is also advisable to delay the arm exercises and physiotherapy to reduce seroma formation [24]. Other factors which increase post-operative seroma formation include patient’s body weight, hypertension, usage of electrocautery, longer procedure duration, no drainage of surgical bed, or usage of "multiple holes" type drain. Certain modifications or interventions have been associated with a decrease in post-operative seroma formation. These are the usage of ultrasonic scissors or electrothermal bipolar vessel system, tetracycline sclerotherapy, and suction drainage [25].

Limitations

Due to restrictions of time and subjects, a total of 30 patients were studied leading to small sample size. The follow-up time is short. Other factors influencing seroma formation such as intraoperative use of diathermy and post-operative delay in arm exercises were not taken into consideration.

## Conclusions

We can conclude from the study that injection octreotide can be used safely and effectively to decrease the volume and duration of lymphorrhea in patients undergoing MRM with axillary dissection with minimal or no complications and adverse reactions. In addition, it reduces the duration of hospital stay and seroma formation and its related morbidity. So, we recommend octreotide use to possibly reduce the most common complications of MRM, i.e., lymphorrhea and subsequent seroma.

## References

[1] Jemal A, Bray F, Center MM, Ferlay J, Ward E, Forman D (2011). Global cancer statistics. CA Cancer J Clin.

[2] (2006). National Cancer Registry Programme - Indian Council of Medical Research. Consolidated report of the population based cancer registries 2001-2004. https://www.ncdirindia.org/All_Reports/PBCR_2001_04/PBCR_2001_04.pdf.

[3] Miles G, Kelvin F (2010). Surgical techniques in breast cancer. Surgery.

[4] Fisher B, Anderson S, Bryant J (2002). Twenty-year follow-up of a randomized trial comparing total mastectomy, lumpectomy, and lumpectomy plus irradiation for the treatment of invasive breast cancer. N Engl J Med.

[5] Yilmaz KB, Dogan L, Nalbant H, Akinci M, Karaman N, Ozaslan C, Kulacoglu H (2011). Comparing scalpel, electrocautery and ultrasonic dissector effects: the impact on wound complications and pro-inflammatory cytokine levels in wound fluid from mastectomy patients. J Breast Cancer.

[6] Carcoforo P, Soliani G, Maestroni U (2003). Octreotide in the treatment of lymphorrhea after axillary node dissection: a prospective randomized controlled trial. J Am Coll Surg.

[7] Kumar S, Lal B, Misra MC (1995). Post-mastectomy seroma: a new look into the aetiology of an old problem. J R Coll Surg Edinb.

[8] Agrawal A, Ayantunde AA, Cheung KL (2006). Concepts of seroma formation and prevention in breast cancer surgery. ANZ J Surg.

[9] Bonnema J, Ligtenstein DA, Wiggers T, van Geel AN (1999). The composition of serous fluid after axillary dissection. Eur J Surg.

[10] Watt-Boolsen S, Nielsen VB, Jensen J, Bak S (1989). Postmastectomy seroma. A study of the nature and origin of seroma after mastectomy. Dan Med Bull.

[11] Aitken DR, Minton JP (1983). Complications associated with mastectomy. Surg Clin North Am.

[12] Chilson TR, Chan FD, Lonser RR, Wu TM, Aitken DR (1992). Seroma prevention after modified radical mastectomy. Am Surg.

[13] Budd DC, Cochran RC, Sturtz DL, Fouty WJ Jr (1978). Surgical morbidity after mastectomy operations. Am J Surg.

[14] Sabry M, Saleh EA, Kamal AE, Abdel Hamid E (2006). Role of octreotide in control of lymphorrhea after axillary node dissection in mastectomy operations-a randomized controlled study. Egypt J Surg.

[15] Mustonen PK, Härmä MA, Eskelinen MJ (2004). The effect of fibrin sealant combined with fibrinolysis inhibitor on reducing the amount of lymphatic leakage after axillary evacuation in breast cancer. A prospective randomized clinical trial. Scand J Surg.

[16] Maton PN (1989). The use of the long-acting somatostatin analogue, octreotide acetate, in patients with islet cell tumors. Gastroenterol Clin North Am.

[17] Kulber DA, Bacilious N, Peters ED, Gayle LB, Hoffman L (1997). The use of fibrin sealant in the prevention of seromas. Plast Reconstr Surg.

[18] Jeffrey SS, Goodson WH 3rd, Ikeda DM, Birdwell RL, Bogetz MS (1995). Axillary lymphadenectomy for breast cancer without axillary drainage. Arch Surg.

[19] Gilly FN, François Y, Sayag-Beaujard AC, Glehen O, Brachet A, Vignal J (1998). Prevention of lymphorrhea by means of fibrin glue after axillary lymphadenectomy in breast cancer: prospective randomized trial. Eur Surg Res.

[20] Ramos SM, O'Donnell LS, Knight G (1999). Edema volume, not timing, is the key to success in lymphedema treatment. Am J Surg.

[21] Kim WT, Ham WS, Koo KC, Choi YD (2010). Efficacy of octreotide for management of lymphorrhea after pelvic lymph node dissection in radical prostatectomy. Urology.

[22] Ulíbarri JI, Sanz Y, Fuentes C, Mancha A, Aramendia M, Sánchez S (1990). Reduction of lymphorrhagia from ruptured thoracic duct by somatostatin. Lancet.

[23] Kottayasamy Seenivasagam R, Gupta V, Singh G (2013). Prevention of seroma formation after axillary dissection—a comparative randomized clinical trial of three methods. Breast J.

[24] Shamley DR, Barker K, Simonite V, Beardshaw A (2005). Delayed versus immediate exercises following surgery for breast cancer: a systematic review. Breast Cancer Res Treat.

[25] van Bemmel AJ, van de Velde CJ, Schmitz RF, Liefers GJ (2011). Prevention of seroma formation after axillary dissection in breast cancer: a systematic review. Eur J Surg Oncol.

